# Retinopathy predicts stroke but not myocardial infarction in type 2 diabetes: the Fremantle Diabetes Study Phase II

**DOI:** 10.1186/s12933-020-01018-3

**Published:** 2020-03-31

**Authors:** Jocelyn J. Drinkwater, Timothy M. E. Davis, Valentina Hellbusch, Angus W. Turner, David G. Bruce, Wendy A. Davis

**Affiliations:** 1 Medical School, The University of Western Australia, Fremantle Hospital, P. O. Box 480, Fremantle, WA 6959 Australia; 2 Lions Eye Institute, Nedlands, WA Australia; 3 Centre for Ophthalmology and Visual Science, University of Western Australia, Crawley, WA Australia

**Keywords:** Diabetes mellitus, Type 2, Diabetic retinopathy, Stroke, Myocardial infarction

## Abstract

**Background:**

Microangiopathy in type 2 diabetes (T2D) is associated with cardiovascular disease (CVD), but most relevant studies were performed > 10 years ago. CVD risk factor management has since improved. The aim of this study was to determine whether diabetic retinopathy (DR) and its severity increases stroke and myocardial infarction (MI) risk in a contemporary cohort.

**Methods:**

Fremantle Diabetes Study Phase II participants with T2D had DR graded from fundus photography at baseline between 2008 and 2011. Subsequent hospitalizations and mortality for MI or stroke were ascertained through validated data linkage to end-2016. Cox regression modelling identified predictors of first stroke and MI including DR presence and severity.

**Results:**

The 1521 participants with T2D and known DR status (mean age 65.6 years, 52.1% males, median diabetes duration 9.0 years) were followed for a mean of 6.6 years. After excluding those with prior MI/stroke, there were 126 incident MIs among 1393 eligible participants and 53 incident strokes in 1473 eligible participants, respectively. Moderate non-proliferative DR (NPDR) or worse was significantly and independently associated with an increased risk of incident stroke (adjusted hazard ratio 2.55 (95% CI 1.19, 5.47), *p *= 0.016). Retinopathy presence and severity increased the risk of incident MI in unadjusted models (*p *≤ 0.001), but these associations were no longer statistically significant after adjusting for other risk factors.

**Conclusions:**

Moderate NPDR or worse was associated with an increased risk of first stroke in Australians with T2D. Intensified CVD risk factor management should be considered for patients with at least moderate NPDR.

## Background

Diabetic retinopathy (DR) is a microvascular complication characterized by microaneurysms, exudates and hemorrhages [1]. Although largely preventable, it affects about a third of people with diabetes [2] and is a leading cause of vision loss [1]. There is some evidence of a link between microvascular disease and the macrovascular complications of type 2 diabetes (T2D) [3]. However, few studies have specifically assessed the relationship between DR and the individual clinical manifestations of cardiovascular disease (CVD). Most of these have involved only stroke as an outcome, and some lacked robust ascertainment of diabetes type and CVD endpoints. In addition, most were conducted more than 10 years ago [4–11] at a time when CVD risk factor management was becoming more intensive [12] and CVD event rates were declining in people with and without diabetes [13, 14]. In Australia, for example, the incidence of stroke in the general population decreased by 23% between 2001 and 2015 and the rate of acute coronary artery disease events by 39% between 2007 and 2013 [15].

Knowledge of the relationship between the microvascular and macrovascular complications of T2D has important implications for screening and risk factor management [16]. In light of this and the recent marked changes in CVD epidemiology in T2D, the aim of the present study was to determine whether the presence and/or severity of DR is associated with incident stroke and myocardial infarction (MI) in a well characterized, contemporary Australian community-based cohort.

## Methods

### Participants and approvals

The Fremantle Diabetes Study Phase II (FDS2) is a community-based, prospective, observational study involving participants with known diabetes from a zip code defined urban community of approximately 157,000 people surrounding the port of Fremantle in the state of Western Australia (WA). Residents within the catchment area with a physician confirmed diabetes diagnosis, and those who had participated in the Fremantle Diabetes Study Phase I (FDS1) but had moved out of the catchment area, were eligible. Sample characteristics including classification of diabetes type and details of those identified but not recruited have been described previously [17]. Briefly, 4639 people with diabetes were identified and 1668 (36%) recruited together with 64 former FDS1 participants. Of these 1732, 1551 (90%) had clinically diagnosed T2D. The study protocol conformed to the ethical guidelines of the 1975 Declaration of Helsinki.

### Clinical assessment

All FDS2 participants underwent a detailed face-to-face assessment at study entry and biennially [17]. Each included questionnaires covering health care utilization, medical conditions, medication use, and socioeconomic, demographic and lifestyle data. Treatment history, body mass index (BMI), age at diagnosis, nature of initial presentation, case record and/or self-identification, and available serum glutamic acid decarboxylase antibody titers were used to determine diabetes type. Non-insulin treated participants and those ≥ 60 years of age at diagnosis were usually considered to have T2D, as were participants < 60 years of age at diagnosis and taking insulin at the time of study entry but whose first treatment was not insulin [18].

A physical examination was conducted by trained registered nurses according to a standard protocol which included visual acuity measured using a Bailey Lovie chart at a distance of 3 metres in a well-lit room, fundus photography using a Canon CR-DGi Non-Mydriatic Retinal Camera, and electrocardiograms which were assessed for relevant abnormalities including atrial fibrillation. Biochemical tests were performed on fasting blood and first morning urine samples using standard automated methods in a single nationally accredited laboratory [18]. The plasma N-terminal pro-B-type natriuretic peptide concentration (NT-proBNP) was measured by commercial assay with inter-day imprecision ≤ 4.0% at concentrations up to 406 pg/mL and a limit of detection of 5 pg/mL.

### Assessment of diabetic retinopathy

Baseline fundus photographs were assessed independently by a single external grader who was accredited by the Centre for Eye Research Australia, University of Melbourne, and who was blinded to other participant data. Diabetic retinopathy was categorized using the modified Airlie House Classification system for the Early Treatment Diabetic Retinopathy Study (ETDRS). The severity of DR was classified as none, mild non-proliferative diabetic retinopathy (NPDR), moderate NPDR, or severe NPDR or worse. Level 10 was classified as no DR, levels 11–31 were categorized as mild NPDR, levels 41 and 84 were considered moderate NPDR and levels 51–80 and 86 were classified as severe NPDR or worse. For participants who did not have fundus photography performed or had ungradable photographs, further information regarding DR status and severity was sought from hospital records, optometrists and ophthalmologists. Those participants who had no information regarding DR status at baseline were excluded from analysis.

### Ascertainment of stroke and myocardial infarction

The Hospital Morbidity Data Collection and the Registry for Births, Deaths and Marriages together capture all hospitalizations and deaths within the state of Western Australia. These sources were accessed for the FDS2 participants through the Western Australian Data Linkage System (WADLS) [19]. The data were used to determine the stroke and MI status and follow-up time ascertained to end-December 2016 using relevant ICD-9-CM and ICD-10-AM diagnosis and procedure codes (Additional file 1: Table SI). The admission date was considered the date of the event. Those who had had an event prior to participating in FDS2 were excluded from the relevant analysis. Causes of death, based on information provided on the death certificate or by the coroner’s determination of cause of death, were reviewed independently by two study physicians (DGB, TMED) and classified under the system used in the UK Prospective Diabetes Study [20]. In the case of discrepant coding, case notes were consulted and a consensus obtained. Participants assessed to have died due to a cardiac or cerebrovascular event were considered as having the respective event in analysis.

Stroke was further classified into four different subtypes, specifically ischemic, hemorrhagic, intracranial hemorrhage, and unspecified, using ICD coding. To determine accuracy of using ICD codes for ascertaining stroke type, medical notes were compared to the ICD coding for 36 cases (67.9%) where both were available, and all were concordant. In three cases medical records were used to ascertain stroke type as the ICD code used was I64 (stroke not specified as hemorrhage or infarction).

### Statistical analysis

Statistical analyses were conducted using the computer packages IBM SPSS for Windows (Version 25.0 Armonk, NY: IBM Corp) and Stata (Version 15.1, Stat Corp, College Station, Texas, USA). Data are presented as proportions, mean ± SD, geometric mean (SD range) or median [interquartile range]. For independent samples, two-way comparisons for categorical variables were by Fisher’s exact test, for normally or log-normally distributed continuous variables by independent samples *t-*test and for variables not conforming to normal or log-normal distribution by Mann–Whitney *U*-test. A two-tailed significance level of *p *< 0.05 was used throughout the study.

Cox proportional hazards models, excluding DR variables, were used to determine the independent predictors of stroke and MI, separately. Diabetic retinopathy presence and severity were then added to the most parsimonious models to determine if they were independent risk factors for stroke and MI. This method was repeated for specific stroke types. Age was used as the timescale with left truncation of age at study entry. Variables were considered for model entry based on clinical relevance and *p *< 0.20 in bivariate analysis. Variables considered for model entry included age, sex, diabetes duration, diabetes treatment, HbA_1c_, blood pressure, BMI, urinary albumin:creatinine ratio, lipids, hemoglobin and smoking status. Atrial fibrillation was considered in the stroke multivariable analyses, and angina and peripheral arterial disease were considered in the MI analysis. Additional Cox regression analyses were performed (i) including participants with prevalent stroke or MI events at baseline, and (ii) excluding participants with a history of ischemic heart disease, cerebrovascular disease, peripheral arterial disease and atrial fibrillation at baseline. The proportional hazards assumption was checked for each model using Schoenfeld global tests and time varying covariates. Since the FDS2 sample size was fixed, a post hoc calculation was performed for all non-significant results to determine whether there was sufficient power to avoid a type II error.

## Results

### Participant disposition

**Fig. 1:**
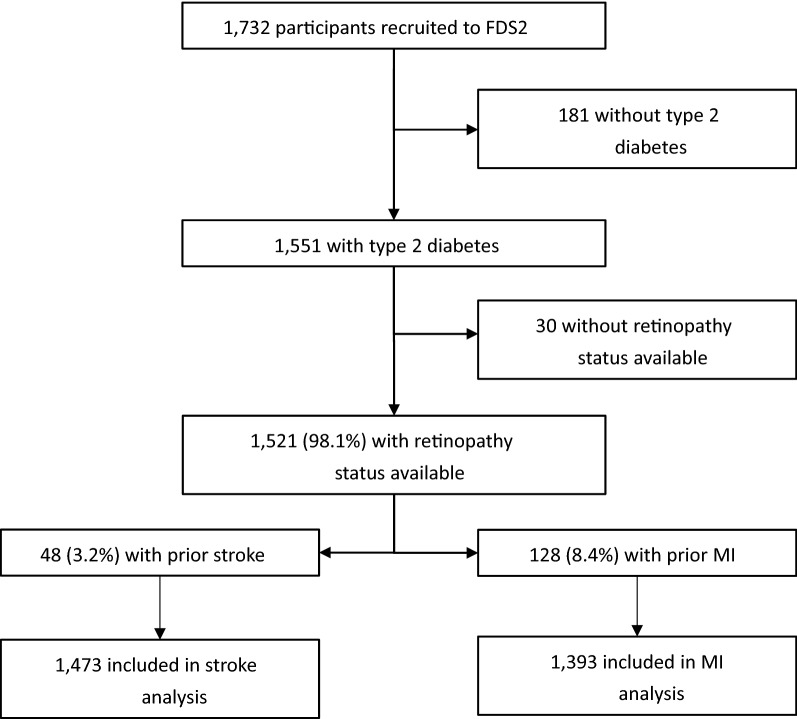
Flow chart showing participants included in analyses

A participant flow diagram is shown in Fig. 1. Of 1551 with T2D, 1418 (91.4%) had gradable fundus photography, whilst 103 (6.6%) had ungradable fundus photography but DR status ascertained from other sources. One participant who had DR documented in the medical record but without classification of severity was conservatively assumed to have mild DR. Diabetic retinopathy status was not available for 30 (1.9%) participants, who were excluded from analyses.

### Baseline characteristics

The 1521 participants with DR status available had a mean ± SD age of 65.6 ± 11.5 years at study entry, 52.1% were male and their median [IQR] diabetes duration was 9.0 [2.9–15.8] years at study entry. There were 565 (37.1%) participants who had DR of whom 444 (29.2%) had mild NPDR, 68 (4.5%) moderate NPDR and 52 (3.4%) severe NPDR or worse. The 48 (3.2%) participants who had suffered a stroke and 128 (8.4%) who had experienced a MI prior to study entry were excluded from the respective analyses.

### Diabetic retinopathy and stroke

During 9759 person-years of follow-up, a mean ± SD follow up of 6.6 ± 1.8 years, there were 53 incident stroke events. Of these 53, there were 24 (45.3%) ischemic, 12 (22.6%) hemorrhagic, 8 (15.1%) intracranial hemorrhages, and 9 (17.0%) unclassified (see Table 1). In bivariate analysis, those who had a stroke during follow-up were older, had longer diabetes duration, a higher HbA_1c_, heart rate and supine systolic blood pressure, and were more likely to be on antihypertensive medication (see Table 2). They were more likely to have atrial fibrillation, poor renal function, anemia, peripheral arterial disease, to have had a MI prior to commencing the study, and to have had an eye examination in the previous year. Those who had a stroke were more likely to have moderate NPDR or worse (7.3% vs 17.0%, *p *= 0.016). However, there was no statistically significant association between any DR or DR severity (as an ordinal variable) at baseline and incident stroke, and a power calculation showed insufficient power to avoid a type II error (< 0.48). Similarly, there was insufficient power to assess the association between retinopathy and the different stroke types separately (see Additional file 1: Table SIV). Therefore, we assessed the association between moderate NPDR or worse with any incident stroke during follow-up.

**Table 1:** The distribution (number (%)) of diabetic retinopathy (DR) and retinopathy severity according to stroke status during follow-up

	No stroke during follow-up	Any stroke during follow-up	Ischaemic stroke	Hemorrhagic stroke	Strokes excluding intracranial hemorrhages
Number	1420	53	24	12	45
Any DR	522 (36.8)	24 (45.3)	12 (50.0)	2 (16.7)	17 (37.8)
DR severity					
None	899 (63.3)	29 (54.7)	12 (50.0)	10 (83.3)	28 (62.2)
Mild NPDR	418 (29.4)	15 (28.3)	7 (29.2)	1 (8.3)	11 (24.4)
Moderate NPDR	61 (4.3)	5 (9.4)	2 (8.3)	0	2 (4.4)
Severe NPDR or PDR	42 (3.0)	4 (7.5)	3 (12.5)	1 (8.3)	4 (8.9)
Moderate NPDR or worse	103 (7.3)	9 (17.0)	5 (20.8)	1 (8.3)	6 (13.3)

**Table 2:** Baseline characteristics of FDS2 participants by stroke status to end-2016

Variables at baseline	No stroke during follow up	Stroke during follow up	*p*-value
Number (%)	1420 (96)	53 (4)	
Age (years)	65.1 ± 11.4	73.0 ± 10.8	< 0.001
Sex (% male)	51.8	52.8	0.89
Ethnic background (%)			0.22
Anglo-Celt	53.7	52.8	
Southern European	12.5	17.0	
Other European	7.5	3.8	
Asian	4.4	5.7	
Indigenous Australian	6.3	0	
Mixed/other	15.6	20.8	
Currently married/de facto (%)	63.6	54.7	0.19
Duration of diabetes (years)	8.3 [2.7–15.6]	12.3 [5.0–17.6]	0.023
Diabetes treatment (%)			0.84
Diet	24.1	20.8	
Oral agents ± non-insulin injectables	53.7	52.8	
Insulin only	5.5	5.7	
Insulin + oral agents ± non-insulin injectables	16.7	20.8	
Fasting glucose (mmol/L)	7.2 [6.2–9.0]	7.2 [6.2–9.3]	0.65
HbA_1c_ (%)	6.8 [6.2–7.7]	7.3 [6.4–8.1]	0.045
HbA_1c_ (mmol/mol)	51 [44–61]	56 [46–65]	0.045
Severe hypoglycemia before baseline (%)	3.0	3.8	0.67
BMI (kg/m^2^)	31.3 ± 6.2	30.2 ± 28.7	0.20
Heart rate (bpm)	70 ± 12	75 ± 16	0.014
Supine SBP (mm Hg)	146 ± 22	155 ± 20	0.004
Supine DBP (mm Hg)	80 ± 12	83 ± 11	0.18
Atrial Fibrillation on ECG (%)	3.5	17.7	< 0.001
Left ventricular hypertrophy on ECG (%)	2.0	0.0	0.62
On antihypertensive medication (%)	72.9	90.6	0.004
On ACE-I	37.7	43.4	0.47
On ARB	32.2	37.7	0.46
On beta-blocker	21.5	30.2	0.13
On calcium channel blocker	23.7	39.6	0.013
On lipid-modifying medication (%)	67.7	77.4	0.18
On aspirin (%)	36.6	47.2	0.15
On other anticoagulant (%)	11.7	20.8	0.05
On digoxin (%)	2.5	7.6	0.048
Total cholesterol (mmol/L)	4.4 ± 1.1	4.3 ± 1.1	0.79
HDL-cholesterol (mmol/L)	1.2 ± 0.3	1.3 ± 0.3	0.71
Serum triglycerides (mmol/L)	1.5 (0.9–2.6)	1.6 (1.0–2.6)	0.35
Urinary albumin:creatinine (mg/mmol)	3.1 (0.8–11.4)	6.7 (1.4–31.5)	< 0.001
eGFR (CKD-EPI) categories (%)			0.001
≥ 90 mL/min/1.73 m^2^	39.8	20.8	
60–89 mL/min/1.73 m^2^	44.8	43.4	
45–59 mL/min/1.73 m^2^	8.5	17.0	
< 45 mL/min/1.73 m^2^	6.9	18.9	
Plasma NT-proBNP (pmol/L)	74 (18–311)	163 (46–582)	< 0.001
Anemia (%)	10.6	20.8	0.039
Ischemic heart disease (%)	28.0	37.7	0.12
Angina before baseline (%)	20.4	26.4	0.30
Myocardial infarction before baseline (%)	8.2	17.0	0.003
Peripheral arterial disease (%)	21.6	35.9	0.018
Peripheral sensory neuropathy (%)	57.5	67.9	0.16
Alcohol (standard drinks/day)	0.1 [0–1.2]	0.1 [0–1.2]	0.95
Smoking status (% never/ex/current)	45.6/43.9/10.5	46.2/46.2/7.7	0.90
Had an eye test in the last year (%)	80.6	93.9	0.015
Any retinopathy (%)	36.8	45.3	0.25
Retinopathy severity (%)			0.06
None	63.3	54.7	
Mild non-proliferative only	29.4	28.3	
Moderate non-proliferative only	4.3	9.4	
Severe non-proliferative or worse	3.0	7.6	
Moderate non-proliferative or worse (%)	7.3	17.0	0.016

Data are percentages, mean ± SD, geometric mean (SD range) or median [inter-quartile range]

The results of the Cox regression are shown in Table 3. Atrial fibrillation, urinary albumin:creatinine ratio and HbA_1c_ were independently associated with stroke in the most parsimonious model. When added to the most parsimonious model, moderate NPDR or worse was associated with an increased stroke risk compared to those with no DR or mild NPDR [HR: 2.55 (95% CI 1.19, 5.47)] and HbA_1c_ was no longer a statistically significant predictor. Schoenfeld global tests and time-varying covariates showed that the proportional hazards assumption was met for these Cox regression models. The results of the additional Cox models are shown in Additional file 1: Tables SV–VII. Severe NPDR was independently associated with incident stroke in those without any prior cardiovascular or cerebrovascular disease [7.13 (1.46, 34.79)]. Moderate NPDR or worse was associated with any stroke when those with a prior stroke at baseline were included [2.21 (1.04, 4.66)].

**Table 3:** Model A shows the most parsimonious Cox model, with age as the timeline, for all incident stroke events. Model B shows the addition of moderate non-proliferative diabetic retinopathy (NPDR) or worse to Model A

Baseline variable	Model A		Model B	
	Cox model, HR (95% CI)	*p*-value	Cox model, HR (95% CI)	*p*-value
HbA_1c_ (per 1% increase)	1.24 (1.03, 1.51)	0.027	1.21 (0.99, 1.47)	0.062
Atrial fibrillation	3.20 (1.50, 6.84)	0.003	3.50 (1.62, 7.56)	0.001
Ln (urinary albumin:creatinine)^a^	1.34 (1.12, 1.61)	0.002	1.30 (1.08, 1.56)	0.006
Moderate NPDR or worse			2.55 (1.19, 5.47)	0.016

^a^A 2.72-fold increase in urinary albumin:creatinine corresponds to an increase of 1 in ln(urinary albumin:creatinine)

### Diabetic retinopathy and myocardial infarction

During 9189 person-years of follow-up, a mean ± SD follow-up of 6.6 ± 1.8 years, there were 126 incident MIs. In bivariate analyses, those who had experienced a MI during follow-up were older, had longer diabetes duration, were more likely to be Indigenous Australians, current smokers, on insulin, have a history of severe hypoglycemia, angina, left ventricular hypertrophy, peripheral arterial disease, peripheral sensory neuropathy, higher systolic blood pressure, albuminuria, and worse kidney function (Additional file 1: Table SII). Incident MI was associated with the presence of any retinopathy (53.2% in those with MI during follow-up vs 34.5% in those with no MI, *p *< 0.001), and greater DR severity (*p *< 0.001).

The results of the Cox regressions for MI are shown in Table 4. In the most parsimonious model (Additional file 1: Table SIII), age at diabetes diagnosis, HbA_1c_, current smoking, ln(plasma NT-proBNP), angina and peripheral arterial disease were independent risk factors for MI (*p *≤ 0.007). Retinopathy status and severity were positively, but non-significantly associated with risk of MI when added to this model (*p *> 0.09). The results of the additional Cox regression that included participants with a prior MI at baseline also showed a significant association in unadjusted models but no independent association (Additional file 1: Table SVIII). There was insufficient power to assess the association between DR and MI in those without any prior cardiovascular or cerebrovascular history at baseline. The proportional hazards assumption was not violated in any of these Cox regressions models.

**Table 4:** The hazard ratios (95% CIs) and significance levels of retinopathy presence and severity for an adjusted Cox model and when added to the most parsimonious Cox model for myocardial infarction with age as the timeline

	Unadjusted model, HR (95% CI)	*p*-value	Most parsimonious model, HR (95% CI)^a^	*p*-value
Any retinopathy	2.10 (1.48, 2.99)	< 0.001	1.30 (0.87, 1.92)	0.197
Retinopathy severity				
None or mild NPDR	1.00 (reference)		1.00 (reference)	
Moderate NPDR	3.15 (1.74, 5.69)	< 0.001	1.73 (0.91, 3.29)	0.095
Severe NPDR or worse	3.11 (1.56, 6.22)	0.001	1.02 (0.48, 2.18)	0.958
Moderate NPDR or worse vs mild NPDR or no DR	2.88 (1.80, 4.58)	< 0.001	1.32 (0.78, 2.25)	0.302

^a^Adjusted for most parsimonious model which comprised age at diabetes diagnosis, HbA1c, current smoker, ln(NT-proBNP), angina and peripheral arterial disease

## Discussion

The present study shows that, in representative, community-based people with T2D, moderate NPDR or worse at baseline was associated with a more than two-fold increase in the risk of any subsequent stroke compared with mild NPDR or no DR after adjustment for a range of other important explanatory variables. Diabetic retinopathy status and its severity were associated with incident MI in bivariate analyses but were not independent risk factors after adjustment for other clinically relevant covariates. This is the first study to assess these relationships in a large contemporary cohort, an important consideration given that CVD epidemiology and risk factor management were much different to when most previous relevant studies were conducted. Although the present findings are consistent with those of most older studies, they have potential implications for current management of T2D and may provide further insight into the pathophysiology of its chronic vascular complications.

### The relationship between diabetic retinopathy and stroke

Several other studies have assessed the relationship between DR and stroke [4, 5, 7–9]. The most adjusted multivariable model from five of these cohort studies involving participants with any type of diabetes [4, 5, 7–9] were included in a recent meta-analysis that found that any DR was significantly associated with an increased risk of stroke (relative risk (RR) 1.74 (95% CI 1.35, 2.24)) [11]. This is lower than the HR of 2.55 found in the present study for moderate NPDR or worse, but the meta-analysis did not include separate analyses based on DR severity. In addition, there were a number of methodologic differences between these and the present studies. The studies in the meta-analysis utilized different definitions and methods of assessing stroke, with three including self-reported stroke [4, 5, 8] and one transient ischemic attacks [7], while one excluded strokes thought to be of cardioembolic origin including those related to atrial fibrillation [9]. By comparison, we ensured that our data were validated and categorized rigorously for both DR and stroke. The World Health Organization Multinational Study of Vascular Diseases in Diabetes (WHO MSVDD) [21], which was not included in the meta-analysis [11], found that any DR was significantly associated with an increased risk of stroke assessed from questionnaires in men and women with T2D (age-adjusted RR 2.1 (95% CI 1.4, 3.2) and 2.4 (1.6, 3.4), respectively) [21]. Although these effect sizes are lower than those of the present study, this may be because we assessed those with worse DR. This suggests a dose response in that increasing DR severity is associated with a higher risk of stroke.

Some studies have shown that the severity of DR is associated with an increased stroke risk. The Action to Control Cardiovascular Risk in Diabetes (ACCORD) trial found an increasing risk of incident fatal or non-fatal stroke during follow-up for each step of DR severity [none, mild, moderate NPDR, to severe NPDR/proliferative DR (PDR)] at baseline [22]. The ACCORD participants with severe NPDR/PDR at baseline had a significantly increased risk of stroke compared to those with no DR (HR 5.57 (95% CI 2.40, 12.96), but this did not apply to those with mild or moderate NPDR [22]. It should be noted, however, that this was a selected sample of patients recruited to a clinical trial. In the Wisconsin Epidemiology Study of Diabetic Retinopathy (WESDR) there was a positive relationship between self-reported stroke and DR severity (OR 1.6 (95% CI 1.1, 2.3) per retinopathy severity step (mild NPDR, moderate to severe NPDR and PDR)) in a type 1 diabetes cohort after 20 years of follow-up [4]. In the older-onset WESDR cohort, a significant relationship was found only between PDR and fatal stroke (HR 1.88 (1.03, 3.43)) after 16 years of follow-up [5]. The Atherosclerosis Risk in Communities Study found a statistically significant relationship between ischemic stroke and any DR [HR: 2.34 (1.13, 4.86)] and also mild to moderate DR [HR: 2.43 (1.32, 4.50)] when compared to the participants with diabetes with no DR, but the association with severe DR was not significant [8]. Taken together with the present results, and notwithstanding between-study differences in patient sources, type of diabetes and stroke ascertainment, there is clear evidence that severe DR is associated with an increased stroke risk.

We were unable to assess the relationship between DR and type of stroke due to the small numbers in each sub-group and therefore a lack of statistical power. However, of interest is that there was a relatively low proportion of ischemic strokes in our cohort. In the Fenofibrate Intervention and Event Lowering in Diabetes (FIELD) trial, 81.4% of first strokes in people with T2D were ischemic and 9.9% hemorrhagic compared with 45.3% and 22.6% in the present study [23]. The FIELD trial data were collected from a younger selected participant sample between 1998 and 2005. Stroke rates have since declined [15] which may reflect a change in stroke distribution away from ischemic events. Indeed, the Global Burden of Disease 2013 Study estimated that 67% of incident strokes worldwide were ischemic and 33% hemorrhagic [24].

### The relationship between diabetic retinopathy and myocardial infarction

The relevant published literature linking DR and MI is inconsistent. Two other studies have similarly found no statistically significant association between DR and MI events, although the WESDR almost reached significance (*p *= 0.06) [4, 25]. The WHO MSVDD found a significant relationship between MI and DR only in type 1 diabetes [21]. The ACCORD trial reported a significant trend between each step of DR severity at baseline and incident fatal or non-fatal MI risk but, compared to those without DR, those with severe NPDR/PDR did not have an increased MI risk [22]. A large population-based cohort study using the UK Clinical Practice Research Datalink found that DR was associated with an increased risk of a major cardiovascular event (defined as either cardiovascular death, non-fatal MI or non-fatal ischemic stroke; HR: 1.39 [95% CI 1.09, 1.76)] [26]. Similarly, the Cardiovascular Health study also showed participants with DR had increased odds of coronary heart disease or stroke [OR: 3.23 (95% CI 1.09, 9.56)] [27]. A meta-analysis reported a significant association between DR and CVD but not specifically MI [10].

Although we did not find a significant independent association between DR and MI, there was a significant association between any DR and DR severity and MI in the unadjusted Cox models. An association between DR and MI is recognised in Europe in that the presence of DR is included in the assessment of cardiovascular risk promoted by the European Society of Cardiology [28]. One explanation for the lack of an independent association between DR and MI in the present study could be that patients with DR may also have autonomic neuropathy. This has been associated with a higher prevalence of asymptomatic MI events [29] which would not have been captured as an outcome in our study as they may not lead to hospitalization. Improved contemporary CVD risk management and increased use of preventive coronary revascularization procedures may have also attenuated an association.

### The association between microvascular and macrovascular disease in diabetes

The link between microvascular and macrovascular complications in diabetes is controversial. A study comprising participants from a diabetes outpatient clinic found that the presence and number of microvascular complications was independently associated with all-cause mortality and cardiovascular events in type 1 diabetes during 10 years of follow-up [30]. The PRECISED study showed that DR presence and severity and microalbuminuria were associated with subclinical cardiovascular disease in people with type 2 diabetes [31]. Retinopathy has also been associated with subclinical carotid atherosclerosis in diabetes [32]. While there are few studies that have assessed the association between diabetes and carotid disease, some have found a significant association [33–36] but others did not [37–40]. Although the Rio de Janeiro type 2 diabetes cohort study found no association between carotid disease and new or worsening DR, participants with carotid disease and microvascular disease at baseline were more likely to have a cardiovascular event than those with carotid disease without microvascular complications [38]. Our results suggest that microvascular disease could be a predictor of macrovascular events as moderate NPDR or worse was independently associated with stroke.

### Potential mechanisms linking diabetic retinopathy and stroke

A potential mechanism linking DR with stroke in T2D is the “common soil” hypothesis in which risk factors including hyperglycemia, hypertension and dyslipidemia are shared by these two complications [41]. Adjustment for these CVD risk factors in the multivariable model did not abolish the relationship between DR and stroke. In addition, when moderate NPDR or worse was added to the most parsimonious model, HbA_1c_ was no longer a significant predictor of stroke. A single baseline measurement of HbA_1c_, providing a measure of glycemia over the previous 3 months, may not adequately capture the effects of chronic glycemic exposure, which may be better represented by the presence of moderate NPDR or worse. In support of this, the weaker association between DR and stroke in our cohort compared with earlier studies could reflect more intensive contemporary long-term CVD risk factor management [13, 14, 42]. The present data suggest that, if DR and especially severe DR is detected on screening, CVD risk factor management should be intensified if necessary and consideration given to other relevant investigations such as carotid ultrasound.

An alternative explanation of the link between DR and stroke is that diabetes-related changes in the retinal microvasculature mirror those in the cerebral microvasculature [43]. In support of this hypothesis, there was a significant association between stroke and severe NPDR or worse when those with any cardiovascular or cerebrovascular disease at baseline were excluded. Cerebral microvascular damage associated with DR could augment the size and thus the neurologic manifestations of cerebral infarction or hemorrhage, thus increasing the likelihood of acute presentation and hospitalization. Recent literature suggests that DR may be a neurodegenerative disease, especially as the breakdown of the blood-retinal-barrier occurs early in the disease process [44] and is more marked in the presence of hyperglycemia [45]. This could also amplify the adverse neurologic effects of an acute intracerebral event. Adding weight to this hypothesis is the observation that, although significant in the unadjusted models, we found no independent association between incident MI and DR after adjustment. This could also reflect the “common soil” hypothesis, with CVD risk factors, even if only measured at baseline, explaining the bivariate relationship without the additional contribution of the close relationship between the retinal and cerebral microcirculations seen in the case of stroke.

### Strengths and limitations

The strengths of the present study include comprehensive assessments in a large community-based sample, independent retinal grading, and near-complete ascertainment of DR status and CVD events using a well-validated data linkage system [19]. Limitations include that as ascertainment of stroke and MI events were by ICD coding through data accessed from WADLS, there is the possibility that some events may have been missed if they occurred outside WA. In addition, we had insufficient power to assess the relationship between DR and different types of stroke.

## Conclusions

The present study found that at least moderate DR is an independent risk factor for stroke but not MI in people with T2D. The retinal microvasculature is readily observable and its appearances should be considered when CVD and especially stroke risk management is planned in T2D.

## Supplementary information

**Additional file 1:** Additional Tables.

## Data Availability

All supporting data are available within this article. Raw data are not publicly available due to restrictions regarding confidentiality, especially with data obtained through the Western Australian Data Linkage System.

## Abbreviations

ACCORD: The Action to Control Cardiovascular Risk in Diabetes
BMI: Body mass index
CVD: Cardiovascular disease
DR: Diabetic retinopathy
ETDRS: Early Treatment Diabetic Retinopathy Study
FDS1: Fremantle Diabetes Study Phase I
FDS2: Fremantle Diabetes Study Phase II
FIELD: Fenofibrate Intervention and Event Lowering in Diabetes
MI: Myocardial infaraction
NPDR: Non-proliferative diabetic retinopathy
NT-proBNP: N-terminal pro-B-type natriuretic peptide
PDR: Proliferative diabetic retinopathy
T2D: Type 2 diabetes
WA: Western Australia
WADLS: West Australian Data Linkage System
WHO MSVDD: World Health Organization Multinational Study of Vascular Diseases in Diabetes
