# Supplementary material for: Retinopathy predicts stroke but not myocardial infarction in type 2 diabetes: the Fremantle Diabetes Study Phase II
Source: Cardiovasc Diabetol. 2020 Mar 31;19:43. doi: 10.1186/s12933-020-01018-3 (PMC7110810; doi:10.1186/s12933-020-01018-3)
Supplement: Supplementary file 1 — Additional file 1: Additional Tables. [file 12933_2020_1018_MOESM1_ESM.docx]

Additional files

Table I: ICD Codes used to determine stroke and myocardial infarction events

|  | Stroke | Myocardial infarction |
| --- | --- | --- |
| ICD-9-CM | 430, 431, 432, 432.0, 432.1, 432.9, 433.01, 433.11, 433.21, 433.31, 433.81, 433.91, 434.01, 434.11, 434.91 | 410, 410.00, 410.01, 410.02, 410.10, 410.104, 10.12, 410.20, 410.21, 410.22, 410.30, 410.31, 410.32, 410.40, 410.41, 410.42, 410.50, 410.51, 410.52, 410.70, 410.71, 410.72, 410.80, 410.81, 410.82, 410.9, 410.91, 410.92 |
| ICD-10-AM | I60, I60.0, I160.1, I60.2, I60.3, I60.4, I60.5, I60.6, I60.7, I60.8, I60.9, I61, I61.0, I61.1, I61.2, I61.3, I61.4, I61.5, I61.6, I61.8, I61.9, I62, I62.0, I62.1, I62.9, I63, I63.0, I63.1, I63.2, I63.3, I63.4, I63.5, I63.6, I63.8, I63.9, I64 | I21, I21.0, I21.1, I21.2, I213, I21.4, I21.9, I22, I22.0, I22.1, I22.8, I22.9 |

Table II: Baseline characteristics of FDS2 participants by incident MI status to end-2016

| Variables at baseline | No MI during follow up | MI during follow up | *p*-value |
| --- | --- | --- | --- |
| Number (%) | 1267 (91.0) | 126 (9.0) |  |
| Age (years) | 64.8 ± 11.3 | 67.9 ± 12.4 | 0.004 |
| Sex (% male) | 49.7 | 50.0 | >0.99 |
| Ethnic background (%): |  |  | 0.002 |
| Anglo-Celt | 53.1 | 54.8 | 0.78 |
| Southern European | 12.6 | 14.3 | 0.58 |
| Other European | 7.8 | 4.8 | 0.29 |
| Indigenous Australian | 5.2 | 13.5 | 0.001 |
| Mixed/other | 21.2 | 12.7 | 0.027 |
| Currently married/de facto (%) | 64.2 | 50.8 | 0.004 |
| Duration of diabetes (years) | 8.0 [2.0-15.0] | 14.8 [6.0-19.9] | <0.001 |
| Diabetes treatment (%): |  |  | <0.001 |
| Diet | 25.8 | 11.1 |  |
| Oral agents± non-insulin injectables | 53.8 | 54.8 |  |
| Insulin only | 4.6 | 10.3 |  |
| Insulin + oral agents± non-insulin injectables | 15.9 | 23.8 |  |
| Fasting glucose (mmol/L) | 7.2 [6.2-8.9] | 6.9 [5.9-9.6] | 0.76 |
| HbA_1c_ (%) | 6.8 [6.2-7.7] | 6.9 [6.2-8.2] | 0.17 |
| HbA_1c_ (mmol/mol) | 51 [44 -61] | 52 [44-66] | 0.17 |
| Severe hypoglycaemia before baseline (%) | 2.4 | 8.7 | 0.001 |
| BMI (kg/m^2^) | 31.4 ± 6.2 | 30.7 ± 6.3 | 0.25 |
| Heart rate (bpm) | 70 ± 12 | 73 ± 16 | 0.08 |
| Supine SBP (mm Hg) | 145 ± 21 | 152 ± 29 | 0.020 |
| Supine DBP (mm Hg) | 80 ± 12 | 79 ± 14 | 0.42 |
| Orthostatic hypotension (%) | 30.7 | 43.5 | 0.005 |
| Atrial Fibrillation on ECG (%) | 4.1 | 4.8 | 0.64 |
| Left ventricular hypertrophy on ECG (%) | 1.5 | 4.8 | 0.021 |
| On antihypertensive medication (%): | 71.7 | 77.0 | 0.21 |
| On lipid-modifying medication (%) | 66.0 | 69.8 | 0.43 |
| On aspirin (%) | 34.0 | 44.4 | 0.024 |
| On other anticoagulant (%) | 9.1 | 22.2 | <0.001 |
| On medication for angina (%) | 2.9 | 10.0 | 0.001 |
| Total cholesterol (mmol/L) | 4.4 ± 1.1 | 4.5 ± 1.7 | 0.30 |
| HDL-cholesterol (mmol/L) | 1.2 ± 0.3 | 1.2 ± 0.4 | 0.94 |
| Serum triglycerides (mmol/L) | 1.5 (0.9-2.5) | 1.6 (0.9-2.8) | 0.20 |
| Urinary albumin:creatinine (mg/mmol) | 3.0 (0.9-10.4) | 6.3 (1.1-36.6) | <0.001 |
| eGFR (CKD-EPI) categories (%): |  |  | <0.001 |
| ≥90 ml/min/1.73m^2^ | 41.6 | 31.2 |  |
| 60-89 ml/min/1.73m^2^ | 45.0 | 38.4 |  |
| 45-59 ml/min/1.73m^2^ | 7.8 | 14.4 |  |
| <45 ml/min/1.73m^2^ | 5.7 | 16.0 |  |
| NTproBNP (pmol/L) | 65 (17-252) | 187 (36-976) | <0.001 |
| Anemia (%) | 8.6 | 20.6 | <0.001 |
| Stroke before baseline (%) | 3.0 | 5.6 | 0.18 |
| Any history of angina at baseline (%) | 14.8 | 37.3 | <0.001 |
| Ischemic heart disease before baseline (%) | 20.6 | 44.4 | <0.001 |
| Peripheral arterial disease (%) | 20.2 | 40.5 | <0.001 |
| Peripheral sensory neuropathy (%) | 56.4 | 65.9 | 0.047 |
| Alcohol (standard drinks/day) | 0.1 [0-1.2] | 0.1 [0-1.2] | 0.40 |
| Smoking status (% never/ex/current) | 47.7/42.9/9.4 | 38.9/42.1/19.0 | 0.004 |
| Had an eye test in the last year (%) | 81.0 | 81.9 | 0.90 |
| Any retinopathy (%) | 34.5 | 53.2 | <0.001 |
| Retinopathy severity (%): |  |  | <0.001 |
| None | 65.6 | 46.8 |  |
| Mild non-proliferative | 28.0 | 35.7 |  |
| Moderate non-proliferative | 3.7 | 10.3 |  |
| Severe non-proliferative or proliferative | 2.7 | 7.1 |  |
| Moderate non-proliferative or worse (%) | 6.4 | 17.5 | <0.001 |

Data are percentages, mean ± SD, geometric mean (SD range) or median [inter-quartile range]

Table III: The most parsimonious Cox model showing independent predictors for incident MI events with age at census as the timeline.

| Baseline Variable | Cox model,  HR (95% CI) | *p*-value |
| --- | --- | --- |
| Age at diabetes diagnosis (increase of 1 year) | 0.97 (0.95, 0.99) | 0.001 |
| HbA_1c_ (per 1% increase) | 1.18 (1.05, 1.34) | 0.007 |
| Current smoker | 2.14 (1.30, 3.51) | 0.003 |
| Ln (NT-proBNP (pmol/L))* | 1.39 (1.23, 1.57) | <0.001 |
| Angina | 2.02 (1.37, 2.98) | <0.001 |
| Peripheral arterial disease | 1.76 (1.20, 2.57) | 0.004 |

* A 2.72-fold increase in x corresponds to an increase of 1 in ln (NT-proBNP (pmol/L))

Table IV: The hazard ratios (95% CIs) and significance levels of different incident stroke types by retinopathy presence and severity when added to the most parsimonious Cox model with age as the timeline. Note that there was insufficient power for these analyses.

| Stroke Type | Ischemic Stroke ^a^ | Hemorrhagic Stroke ^b^ | All stroke excluding intracranial hemorrhages ^c^ |
| --- | --- | --- | --- |
| Any retinopathy | 1.13 (0.49, 2.63), *p*=0.77 | 0.36 (0.08, 1.68), *p*=0.20 | 0.79 (0.41, 1.52), *p*=0.47 |
| Retinopathy severity: |  |  |  |
| None or mild NPDR | 1.00 (reference category) | 1.00 | 1.00 |
| Moderate NPDR | 1.72 (0.39, 7.58) *p*=0.47 | N/A* | 1.10 (0.26, 4.75), *p*=0.90 |
| Severe NPDR or PDR | 3.32 (0.90, 12.3), *p*=0.07 | 1.80 (0.19, 17.33), *p*=0.61 | 2.16 (0.70, 6.72), *p*=0.18 |
| Moderate NPDR or worse (vs mild NPDR or no DR) | 2.38 (0.84, 6.75), *p*=0.10 | 1.13 (0.13, 9.63), *p*=0.91 | 1.61 (0.63, 4.14), *p*=0.32 |

^a^Variables in the most parsimonious Cox model for ischemic stroke: HbA_1c_ and urinary albumin:creatinine ratio; ^b^Variables in the most parsimonious Cox model for hemorrhagic stroke: atrial fibrillation and estimated glomerular filtration rate; ^c^Variables in the most parsimonious Cox model for all stroke excluding intracranial hemorrhages: HbA_1c_, atrial fibrillation, urinary albumin:creatinine ratio and peripheral arterial disease; *No participants with moderate NPDR had a hemorrhagic stroke

Table V: The relationship between retinopathy and any stroke in unadjusted and adjusted Cox regression models after excluding those participants with any history of cardiovascular disease, cerebrovascular disease, angina, ischaemic heart disease, peripheral arterial disease or atrial fibrillation at baseline. There were 791 participants included and 17 (2.1%) stroke events during follow-up after these exclusions.

|  | Unadjusted model, HR (95% CI) | *p-*value | Most parsimonious model, HR (95% CI)* | *p*-value |
| --- | --- | --- | --- | --- |
| Any retinopathy^†^ | 2.45 (0.91, 6.62) | 0.077 | 2.19 (0.79, 6.07) | 0.130 |
| Retinopathy severity: |  |  |  |  |
| None or mild NPDR | 1.00 (reference) |  | 1.00 (reference) |  |
| Moderate NPDR | 2.13 (0.26, 17.17) | 0.477 | 1.88 (0.23, 15.15) | 0.553 |
| Severe NPDR or worse | 10.41 (2.26, 47.96) | 0.003 | 7.48 (1.53, 36.68) | 0.013 |
| Moderate NPDR or worse  (vs mild NPDR or no DR) | 4.68 (1.28, 17.05) | 0.019 | 3.75 (0.99, 14.25) | 0.052 |
| Severe NPDR or worse (vs less than severe NPDR) | 10.05 (2.19, 46.10) | 0.003 | 7.13 (1.46, 34.79) | 0.015 |

*Adjusted for most parsimonious model which comprised macroalbuminuria (HR: 6.69 (95%CI:2.05-21.81), *P*=0.002). †Insufficient power for analysis

Table VI: The relationship between retinopathy and any stroke in unadjusted and adjusted Cox regression models including those who had a stroke before baseline. There were 1521 participants with type 2 diabetes and retinopathy status available. Of these, there were 58 (3.8%) stroke events after baseline.

|  | Unadjusted model, HR (95% CI) | *p-*value | Most parsimonious model, HR (95% CI)* | *p*-value |
| --- | --- | --- | --- | --- |
| Any retinopathy^†^ | 1.30 (0.77, 2.18) | 0.330 | 1.14 (0.66, 1.99) | 0.634 |
| Retinopathy severity: |  |  |  |  |
| None or mild NPDR | 1.00 (reference) |  | 1.00 (reference) |  |
| Moderate NPDR | 2.23 (0.88, 5.63) | 0.089 | 2.39 (0.92, 6.17) | 0.073 |
| Severe NPDR or worse | 3.25 (1.16, 9.09) | 0.025 | 2.00 (0.68, 5.89) | 0.207 |
| Moderate NPDR or worse  (vs mild NPDR or no DR) | 2.59 (1.27, 5.30) | 0.009 | 2.205 (1.04, 4.66) | 0.038 |
| Severe NPDR or worse (vs less than severe NPDR) | 3.08 (1.10, 8.60) | 0.032 | 2.00 (0.68, 5.89) | 0.207 |

*Adjusted for the most parsimonious model which comprised HbA1c, atrial fibrillation and ln(urinary albumin:creatinine). ^†^Insufficient power (14%)

Table VII: Model A shows the most parsimonious Cox model, with age as the timeline, for all stroke events after baseline, regardless of whether participants had a stroke before study entry, as per Table VI. Model B shows the addition of moderate non-proliferative diabetic retinopathy (NPDR) or worse to Model A.

| Baseline Variable | Model A | | Model B | |
| --- | --- | --- | --- | --- |
|  | Cox model, HR (95% CI) | *p*-value | Cox model, HR (95% CI) | *p*-value |
| HbA_1c_ (per 1% increase) | 1.24 (1.03, 1.50) | 0.026 | 1.22 (1.00, 1.48) | 0.049 |
| Atrial fibrillation | 3.31 (1.65, 6.64) | 0.003 | 3.42 (1.79, 6.90) | 0.001 |
| Ln (urinary albumin:creatinine)* | 1.32 (1.11, 1.58) | 0.002 | 1.29 (1.08, 1.55) | 0.005 |
| Moderate NPDR or worse |  |  | 2.21 (1.04, 4.66) | 0.038 |

* A 2.72-fold increase in urinary albumin:creatinine corresponds to an increase of 1 in ln(urinary albumin:creatinine)

Table VIII: The relationship between retinopathy and any MI in unadjusted and adjusted Cox regression models including those who had a MI before baseline. There were 1521 participants with type 2 diabetes and retinopathy status available. Of these, 168 (11.0%) had a MI after baseline.

|  | Unadjusted model, HR (95% CI) | *p-*value | Most parsimonious model, HR (95% CI)* | *p*-value |
| --- | --- | --- | --- | --- |
| Any retinopathy | 2.24 (1.65, 3.03) | <0.001 | 1.40 (0.99, 1.97) | 0.058 |
| Retinopathy severity: |  |  |  |  |
| None or mild NPDR | 1.00 (reference) |  | 1.00 (reference) |  |
| Moderate NPDR | 2.90 (1.76, 4.77) | <0.001 | 1.57 (0.91, 2.73) | 0.107 |
| Severe NPDR or worse | 3.15 (1.77, 5.61) | <0.001 | 1.08 (0.57, 2.04) | 0.818 |
| Moderate NPDR or worse  (vs mild NPDR or no DR) | 3.00 (2.02, 4.45) | <0.001 | 1.33 (0.84, 2.11) | 0.227 |

*Adjusted for the most parsimonious model which comprised age at diabetes diagnosis, HbA1c, peripheral arterial disease, anemia, ln(NTproBNP), angina and current smoker.
